# Use of AI Language Engine ChatGPT 4.0 to Write a Scientific Review Article Examining the Intersection of Alzheimer’s Disease and Bone

**DOI:** 10.1007/s11914-023-00853-z

**Published:** 2024-01-16

**Authors:** Tyler J. Margetts, Sonali J. Karnik, Hannah S. Wang, Lilian I. Plotkin, Adrian L. Oblak, Jill C. Fehrenbacher, Melissa A. Kacena, Alexandru Movila

**Affiliations:** 1 Department of Orthopaedic Surgery, Indiana University School of Medicine, Indianapolis, IN 46202 USA; 2 Department of Anatomy, Cell Biology & Physiology, Indiana University School of Medicine, Indianapolis, IN 46202 USA; 3 Indiana Center for Musculoskeletal Health, Indiana University School of Medicine, Indianapolis, IN USA; 4 Richard L. Roudebush VA Medical Center, Indianapolis, IN 46202 USA; 5 Department of Radiology & Imaging Sciences, Stark Neurosciences Research Institute, Indiana University School of Medicine, Indianapolis, IN 46202 USA; 6 Stark Neurosciences Research Institute, Indiana University School of Medicine, Indianapolis, IN 46202 USA; 7 Department of Pharmacology and Toxicology, Indiana University School of Medicine, Indianapolis, IN 46202 USA; 8 Department of Biomedical Sciences and Comprehensive Care, Indiana University School of Dentistry, Indianapolis, IN 46202 USA

**Keywords:** Artificial intelligence, AI, ChatGPT, Scientific writing, Alzheimer’s Disease, Osteoporosis, Fractures, Bone loss

## Abstract

**Purpose of Review:**

This Comment represents three review articles on the relationship between Alzheimer’s disease, osteoporosis, and fracture in an exploration of the benefits that AI can provide in scientific writing. The first drafts of the articles were written (1) entirely by humans; (2) entirely by ChatGPT 4.0 (AI-only or AIO); and (3) by humans and ChatGPT 4.0 whereby humans selected literature references, but ChatGPT 4.0 completed the writing (AI-assisted or AIA). Importantly, each review article was edited and carefully checked for accuracy by all co-authors resulting in a final manuscript which was significantly different from the original draft.

**Recent Findings:**

The human-written article took the most time from start to finish, the AI-only article took the least time, and the AI-assisted article fell between the two. When comparing first drafts to final drafts, the AI-only and AI-assisted articles had higher percentages of different text than the human article. The AI-only paper had a higher percentage of incorrect references in the first draft than the AI-assisted paper. The first draft of the AI-assisted article had a higher similarity score than the other two articles when examined by plagiarism identification software.

**Summary:**

This writing experiment used time tracking, human editing, and comparison software to examine the benefits and risks of using AI to assist in scientific writing. It showed that while AI may reduce total writing time, hallucinations and plagiarism were prevalent issues with this method and human editing was still necessary to ensure accuracy.

**Supplementary Information:**

The online version contains supplementary material available at 10.1007/s11914-023-00853-z.

## Introduction

Time is invaluable, and the advancements of artificial intelligence (AI) provide new avenues to save this precious resource. Attention to this field increased dramatically with the introduction of ChatGPT by OpenAI in November of 2022. As ChatGPT has been used to improve the quality and speed of many human tasks, such as coding and translation, one could also expect the language model to have potential benefits in writing a scientific review article [1]. With its vast knowledge base and ability to search through text at incredible speeds, usage of ChatGPT should be able to reduce the time required to do a comprehensive literature search. Additionally, it is a skilled language program that can assist with grammar, vocabulary, and writing style. With this background, we sought to compare three writing strategies to evaluate whether AI utilization can save time in the composition of a scientific review article. The topic of all review articles assessed in this Comment was “The Intersection of Alzheimer’s Disease and Bone” [2–4]. The first draft of the first article was written entirely by a human in the traditional method of writing a review article [2]. The first draft of the second article was written entirely by ChatGPT with human-written queries (AI-only or AIO) [3]. In the third article, the text of the references gathered for the first article was provided to ChatGPT, and then ChatGPT was prompted with human-written queries to write the first draft using the provided references (AI-assisted or AIA) [4]. Here we analyze a number of quantifiable metrics including the amount of human editing required for each paper. We also compared references from the first and last drafts to evaluate levels of improper citations and hallucinations. Please see the *Introductory Comment* [5] for more details regarding study design.

Here we hypothesized that while the AIO and AIA papers would require less time to write, they would involve more editing and the AIO paper would have greater differences in references between first and final drafts. Additionally, plagiarism data of first drafts was compared, with the expectation that both AIO and AIA drafts would have more plagiarism than the human paper. It should be noted that we followed this same process for two additional topic areas related to the scope of *Current Osteoporosis Research*: “Neural Regulation of Fracture Healing” [6–9] and “COVID-19 and Musculoskeletal Health” [10–12].

The first drafts for human-written, AIO, and AIA review articles about the intersection of Alzheimer’s disease and bone are included in Supplemental Materials 1–3, respectively. All queries and AI responses used to generate the first drafts of the AIO and AIA review articles are included in Supplemental Materials 4 and 5, respectively. ChatGPT was used to suggest ideas for graphical abstracts for these papers, which were then produced using BioRender. Queries and AI responses for AIO and AIA graphical abstracts are included in Supplemental Materials 6 and 7, respectively. The queries and responses to select and annotate important references for the AIO and AIA papers are included in Supplemental Materials 8 and 9, respectively.

## Results

Table 1 shows the total time taken to produce a review article of publishable quality with each writing method on the topic “The Intersection of Alzheimer’s Disease and Bone.” The time was categorized by different steps within the writing process. The time required for outline and literature review for the human paper was attributed to the AIA paper as well, since the same outline and resources were utilized to write the AIA paper. In total, the AIO paper took the least amount of time at 35.63 total hours, followed by the AIA paper at 64.89 h, and the human paper at 78.03 h.

**Table 1:** The total time in hours for each of “The Intersection of Alzheimer’s Disease and Bone” review articles differentiated into type of activity

Activity	Human	AI-only	AI-assisted
Preparation (h)	0	5.83	6.20
Literature review (h)	13.93	0	13.93
Outline (h)	0.33	0.25	0.33
Writing (h)	29.25	1.70	9.17
Fact checking AI (h)	0	8.35	7.08
Student edits (h)	16.38	7.42	16.75
Faculty edits (h)	16.50	10.83	10.68
Other (h)	1.64	1.25	0.75
Total time (h)	78.03	35.63	64.89

The first draft of the human paper was written in the traditional method, while the first drafts of the AIO and AIA papers were completely synthesized by ChatGPT in response to queries written by human authors. The comparison of similarity between these first drafts and the final drafts is shown in Table 2. The human paper showed the least percentage of text that was explicitly different at 9.5%. The AIO and AIA papers differed by 26.5% and 25.6%, respectively. The remaining percent of the papers was divided into three categories of similarity: identical, minor changes, and paraphrased. Between the three papers, the human paper had the highest percent of identical text at 30.9%, AIO had the highest percent of minorly changed text at 44.6%, and AIA had the highest percent of paraphrased text at 28.5%.

**Table 2:** The similarity percentages between first and final drafts of “The Intersection of Alzheimer’s Disease and Bone” review articles

Level of similarity	Human	AI-only	AI-assisted
Identical (%)	30.9	9.4	23.0
Minor changes (%)	37.4	44.6	22.9
Paraphrased (%)	22.2	19.5	28.5
Different (%)	9.5	26.5	25.6

All queries to create the first drafts of the AIO and AIA papers are included in Supplemental Materials 4 and 5. In total, ChatGPT was queried 28 times while writing the AIO paper, and 100 times for writing of the AIA paper. While writing the first drafts in this method, it became apparent that the text produced by ChatGPT had numerous errors and inaccurate references that would have taken an onerous number of queries to correct. For this reason, all edits beyond the first draft were performed by humans for both papers.

The number of correct and incorrect AI-generated references in the first drafts of each AI paper is shown in Table 3. References were considered incorrect if they were (1) the wrong year or author, (2) referenced the wrong information, or (3) were completely fabricated. AIO had an incorrect reference percentage of 44.2%, while AIA had 8.6%.

**Table 3:** The number of AI references that were correct in first drafts of “The Intersection of Alzheimer’s Disease and Bone” review articles

Reference criteria	AI-only	AI-assisted
AI-cited references	43	70
Correct AI-cited references	24	64
Incorrect AI references	19	6
Incorrect reference percentage	44.2%	8.6%

Table 4 shows the similarity index (plagiarism detection) of the first and final drafts of each paper as generated by the Turnitin software. The AIA paper had the highest first draft similarity index at 36%, followed by the human paper at 10% and the AIO paper at 9%. While editing caused a zero and 3% difference in the similarity scores of the human and AIO papers, respectively, the final draft of the AIA paper had a 19% similarity index, a difference of 17% from the first draft.

**Table 4:** Plagiarism similarity index for the first and final drafts for “The Intersection of Alzheimer’s Disease and Bone” review articles

	Human	AI only	AI assisted
Plagiarism similarity index first draft (%)	10	9	36
Plagiarism similarity index final draft (%)	10	6	19

## Discussion

The main goal of this writing experiment was to evaluate the ability of AI to save time during the process of writing a review article. While all articles related to the Comment were focused on the topic “The Intersection of Alzheimer’s Disease and Bone,” and therefore contained similar information, they were all unique and differed greatly in the process of reaching publishable quality [2–4]. A majority of the time spent on the human paper was spent during the literature review and writing phase. This was to be expected, however, as it takes a considerable amount of time to gather resources, read articles, and incorporate large amounts of information together in an appropriate manner. This style of writing also requires the authors to spend a considerable amount of time ensuring the article has proper grammar, word choice, and structure. These factors led to a total of 29.25 h spent in the writing phase of the human-written article. In comparison, the AIA article required 9.17 h to write and the AIO article required a mere 1.7 h. The AIA article required the author to convert resources into a format readable by ChatGPT, input sources one by one, and work through errors encountered while using the software. This increased the writing time of this article with respect to the AIO article, but still did not approach the human article time. The discrepancy in writing time between human and AI articles shows that AI could be a valuable time-saving tool during this phase of preparing a review article, especially for writers who are less proficient in English vocabulary and writing techniques.

Although the writing time was much lower for AI-generated articles, additional total time was added during the “fact checking” process. Through initial trials, our groups were able to verify that ChatGPT tends to misappropriate information to incorrect citations and occasionally fabricates resources. This required a thorough fact check of both the AIO and AIA papers that added similar levels of time to the totals, at 8.35 and 7.08 h, respectively. This must be considered when contemplating the use of AI in writing a review article, as accuracy of information cannot be jeopardized in the pursuit of speed.

It is important to note that the various manuscripts were “written” by authors having different years of experience in writing scientific manuscripts as well as familiarity with the subject knowledge. The first drafts of the human and AIA papers were composed by medical students with little experience in the field, while the first draft of the AIO paper was written by a research faculty member with experience in scientific manuscript writing and familiarity with the subject matter. The experience of the author in scientific writing might have helped reduce the time required for editing and fact checking in the AIO-based model. Ultimately, subject familiarity would qualify authors to be able to judge the quality of work generated by AI: experienced authors would be able to recognize and edit overly ornamental language, inaccurate references, and overreaching statements. Recognizing these deficits would be more difficult for a novice in the field (such as a medical student or other graduate student) and may have contributed to differences in total times between the AIO and other manuscripts. On the flip side, the AIO underwent intense fact checking and rewriting of entire subsections to make a good publishable review. This involved efforts beyond the first author (research faculty), including expert faculty and medical students. It can be inferred that even though the AIO article took less time to generate a publishable manuscript, the process was still labor intensive. The AIA article took less time overall than the human-written article, and the first authors of those manuscripts have very similar levels of experience in the field. The AIA approach also required a good amount of editing by the co-authors, especially content experts. It is important for AI users to keep these factors in mind when writing scientific research or review articles, as the time-saving benefits of AI are likely relative to the author’s capability to use the software efficiently as well as demonstrate a high level of expertise in the scientific area.

When comparing the first and final drafts of the human-written article, there was a much lower percentage of explicitly different text compared to the AI-written articles. In the AIO article, this may be attributed to the fact that AI did not include sufficient information and resources required for a comprehensive review of the topic. As a result, this information had to be manually inserted during the editing phase. It could be expected that this problem would be alleviated by feeding ChatGPT the exact resources that are needed to write a comprehensive review; however, AI does not always include the information that the author desires to use from a specific article. Manual inclusion of new information in this article led to a similar percentage of different information to what was seen in the AIO article. Authors must consider that altering queries can be helpful in obtaining desired information out of AI writing, but with the current versions of the software it is very likely that all drafts will ultimately require human intervention to be of publishable quality.

Nearly half of the AIO references produced for the first draft were incorrect and unusable, requiring thorough fact checking and knowledge of the literature to create an appropriate draft. The AIA paper produced incorrect references at a much lower percentage, likely because ChatGPT was not asked to list authors and dates of references, only to refer to them by their document ID number given by the PDF plugin. Even so, the AIA paper associated document ID numbers with the wrong information on 6 occasions, requiring thorough fact checking for this paper as well. The current AI software is excellent at sifting through endless pages of text to generate ideas and synthesize accurate paragraphs but seems to struggle with the concept of proper citation. It appears this issue can be alleviated through use of an AIA model that provides specific resources to the software, but it is likely that this method increases production time while still requiring a fact check of the software’s writing.

As expected, the plagiarism similarity index of the AIA paper was higher than the human-written paper, at 36% and 10%, respectively. This percentage represents the amount of text in the first draft that was highly similar in wording to internet resources and published articles. Turnitin divides the scoring into five categories, with blue having no matching text, green with 1–24%, yellow 25–49%, orange 50–74%, and red 75–100% matching text. It was discovered through initial queries in the AIA paper that ChatGPT would write with wording very similar to articles that it was asked to analyze, and this finding was confirmed through the similarity score in the yellow range which is highly concerning for plagiarism. Interestingly, the first draft of the AIO paper had a score of 9%, slightly less than the human-written paper. This score falls in the green range and represents a much lower percentage of similar text than the AIA paper, suggesting that prompting AI to write without giving it explicit sources to use results in less plagiarism. Plagiarism similarity scores of the final drafts of the human and AIO papers were nearly identical to their respective first draft scores. This may be attributed to the low starting scores, as both first drafts started well within the green zone and thus did not require editing of highly similar wording. The AIA paper required much more effort to bring the similarity score into the green zone. While AI-plagiarized phrases can and should be edited to become original writing, this is time consuming and can be overlooked if proper plagiarism detection software is not used.

Overall, the results of our writing experiment support the notion that the use of AI can expedite the lengthy process of writing a scientific review article. Users must be extremely thorough in the editing phase, as AI will hallucinate facts and cite resources incorrectly when responding to human queries. While AI is not yet a reliable way to produce scientific literature on its own, it provides a promising avenue to save our precious time.

### Supplementary Information

Below is the link to the electronic supplementary material.

Supplementary file1 (DOCX 445 KB)

## Data Availability

No datasets were generated or analysed during the current study.

## References

[1] Huang J, Tan M (2023). The role of ChatGPT in scientific communication: writing better scientific review articles. Am J Cancer Res.

[2] Wang HS, Karnik SJ, Margetts TJ, et al. Mind gaps & bone snaps: exploring the connection between Alzheimer’s disease & osteoporosis. Curr Osteoporos Rep. 2024. 10.1007/s11914-023-00851-1 10.1007/s11914-023-00851-1 PMC11420299 38236512

[3] Karnik SJ, Margetts TJ, Wang HS, et al. Mind the gap: unraveling the intricate dance between Alzheimer’s disease and related dementias and bone health. Curr Osteoporos Rep. 2024. 10.1007/s11914-023-00847-x 10.1007/s11914-023-00847-x PMC10912190 38285083

[4] Margetts TJ, Wang HS, Karnik SJ, et al. From the mind to the spine: the intersecting world of Alzheimer’s and osteoporosis. Curr Osteoporos Rep. 2024. 10.1007/s11914-023-00848-w 10.1007/s11914-023-00848-w PMC10912148 38334917

[5] Kacena MA, Plotkin LI, Fehrenbacher JC. The use of artificial intelligence in writing scientific review articles. Curr Osteoporos Rep. 2024. 10.1007/s11914-023-00852-0 10.1007/s11914-023-00852-0 PMC10912250 38227177

[6] Nazzal MK, Morris AJ, Parker RS, et al. Don’t lose your nerve, be callus: insights into neural regulation of fracture healing. Curr Osteoporos Rep. 2024. 10.1007/s11914-023-00850-2 10.1007/s11914-023-00850-2 PMC10912323 38294715

[7] Morris AJ, Parker RS, Nazzal MK, et al. Cracking the code: the role of peripheral nervous system signaling in fracture repair. Curr Osteoporos Rep. 2024. 10.1007/s11914-023-00846-y 10.1007/s11914-023-00846-y PMC10912155 38236511

[8] Parker RS, Nazzal MK, Morris AJ, et al. Role of the neurologic system in fracture healing: an extensive review. Curr Osteoporos Rep. 2024. 10.1007/s11914-023-00844-0 10.1007/s11914-023-00844-0 PMC10912173 38236509

[9] Nazzal MK, Morris AJ, Parker RS, et al. Using AI to write a review article examining the role of the nervous system on skeletal homeostasis and fracture healing. Curr Osteoporos Rep. 2024. 10.1007/s11914-023-00854-y 10.1007/s11914-023-00854-y PMC10912134 38217755

[10] Creecy A, Awosanya OD, Harris A, et al. COVID-19 and bone loss: a review of risk factors, mechanisms, and future directions. Curr Osteoporos Rep. 2024. 10.1007/s11914-023-00842-2 10.1007/s11914-023-00842-2 PMC10912142 38221578

[11] Harris A, Creecy A, Awosanya OD, et al. SARS-CoV-2 and its multifaceted impact on bone health: mechanisms and clinical evidence. Curr Osteoporos Rep. 2024. 10.1007/s11914-023-00843-1 10.1007/s11914-023-00843-1 PMC10912131 38236510

[12] Awosanya OD, Harris A, Creecy A, et al. The utility of AI in writing a scientific review article on the impacts of COVID-19 on musculoskeletal health. Curr Osteoporos Rep. 2024. 10.1007/s11914-023-00855-x 10.1007/s11914-023-00855-x PMC10912275 38216806

